# Predictors for mortality from respiratory failure in a general population

**DOI:** 10.1038/srep26053

**Published:** 2016-05-16

**Authors:** Maki Kobayashi, Yoko Shibata, Sumito Inoue, Akira Igarashi, Kento Sato, Masamichi Sato, Takako Nemoto, Yuki Abe, Keiko Nunomiya, Michiko Nishiwaki, Yoshikane Tokairin, Tomomi Kimura, Makoto Daimon, Naohiko Makino, Tetsu Watanabe, Tsuneo Konta, Yoshiyuki Ueno, Takeo Kato, Takamasa Kayama, Isao Kubota

**Affiliations:** 1 Department of Cardiology, Pulmonology, and Nephrology, Yamagata University School of Medicine, Yamagata, Japan; 2 Global Center of Excellence Program Study Group, Yamagata University School of Medicine, Yamagata, Japan

## Abstract

Risk factors for death from respiratory failure in the general population are not established. The aim of this study was to determine the characteristics of individuals who die of respiratory failure in a Japanese general population. In total, 3253 adults aged 40 years or older participated in annual health check in Takahata, Yamagata, Japan from 2004 to 2006. Subject deaths through the end of 2010 were reviewed; 27 subjects died of respiratory failure (pneumonia, n = 22; COPD, n = 1; pulmonary fibrosis, n = 3; and bronchial asthma, n = 1). Cox proportional hazard analysis revealed that male sex; higher age, high levels of D-dimer and fibrinogen; lower body mass index (BMI) and total cholesterol; and history of stroke and gastric ulcer were independent risk factors for respiratory death. On analysis with C-statistics, net reclassification improvement, and integrated discrimination improvement, addition of the disease history and laboratory data significantly improved the model prediction for respiratory death using age and BMI. In conclusion, we identified risk factors for mortality from respiratory failure in a prospective cohort of a Japanese general population. Men who were older, underweight, hypocholesterolemic, hypercoagulo-fibrinolytic, and had a history of stroke or gastric ulcer had a higher risk of mortality due to respiratory failure.

Respiratory failure is a common cause of death worldwide. According to the World Health Organization, pneumonia and chronic obstructive pulmonary disease (COPD) were the third and fourth leading causes of death in 2004, respectively^1^. Therefore, decreasing the number of deaths caused by respiratory failure is important for improving the health of the general population.

We previously demonstrated that a reduced forced expiratory volume in 1 s (FEV1) is associated with mortality due to all causes and cardiovascular disease specifically in a Japanese general population^2^. However, although low FEV1 was a significant risk factor for death by respiratory failure in a univariate Cox proportional hazard analysis, low FEV1 was not a risk for respiratory death independent of other confounding factors such as age, sex, smoking, blood pressure, liver function, renal function, and dyslipidemia in multivariate analysis^2^. This result suggests that the risk of respiratory death cannot be evaluated solely based on pulmonary function. Hidden factors associated with respiratory death must be identified in order to devise measures to reduce these deaths in the general population.

To date, the risk factors associated with death caused by respiratory failure have not been fully investigated in a general population. The aim of this study was to determine the characteristics of individuals who die by respiratory failure in a Japanese general population.

## Results

### Comparison of characteristics between subject groups

During the 7-year study period, 127 subjects died. Among these, 27 deaths were attributed to respiratory failure as follows: pneumonia (n = 22), COPD (n = 1), pulmonary fibrosis (n = 3), and bronchial asthma (n = 1). The subject population was divided into three groups as follows: subjects who were alive at the end of the study period (group A, n = 3126); those died of respiratory failure (group DR, n = 27); and those who died of diseases other than respiratory failure (group DoR, n = 100). The subject characteristics in the three groups are summarized in Table 1. The mean age, renin activity, and homocysteine (Hcy), D-dimer (D-D), and fibrinogen concentrations in groups DR and DoR were significantly higher than those in group A. The proportion of female subjects was significantly lower in groups DR and DoR than in group A. The Brinkman Index, serum creatinine (sCr), uric acid (UA) concentration, and adiponectin concentration in group DoR were significantly higher than those in group A, but they did not differ significantly between groups DR and A. The body mass index (BMI) and total cholesterol (TC) in group DR were significantly lower than those in groups A and DoR. The red blood cell count (RBC) and FVC % predicted in group DoR was significantly lower than those in group A, but the two variables did not differ significantly between groups DR and A. The FEV1 % predicted in groups DR and DoR was significantly lower than in group A. The percentage of individuals with a FEV1/FVC < 0.7 was significantly higher in groups DR and DoR than in group A. The aspartate aminotransferase (AST) in group DR was significantly higher than that in group A, but did not differ between groups DoR and A. Subjects with a history of stroke, gastric ulcer, and cancer were more frequent in group DR than in groups A and DoR. Subjects with a history of respiratory disease were less frequent in group A compared with groups DR and DoR, but the percentage did not differ between groups DR and DoR.

### Screening of independent risk factors for death by respiratory failure

The independent risk factors for death by respiratory failure were determined using multivariate Cox proportional hazard tests (Table 2). We selected the variables that differed significantly between groups A and DR (*P* < 0.001) based on the laboratory data and past disease history shown in Table 1. Pulmonary function parameters were not included because they were not independent risk factors for respiratory death according to a previous study^2^. Male sex; higher age; high levels of D-D, and fibrinogen; lower BMI and TC; and a history of stroke and gastric ulcer were independent risk factors for respiratory death. However, level of Hcy and a history of cancer and respiratory disease were not independent risks for respiratory death in this study population. There was no multicollinearity among the covariates in this analysis (data not shown).

### Cut-off values of risk factors and Kaplan-Meier analyses

**Figure 1:**
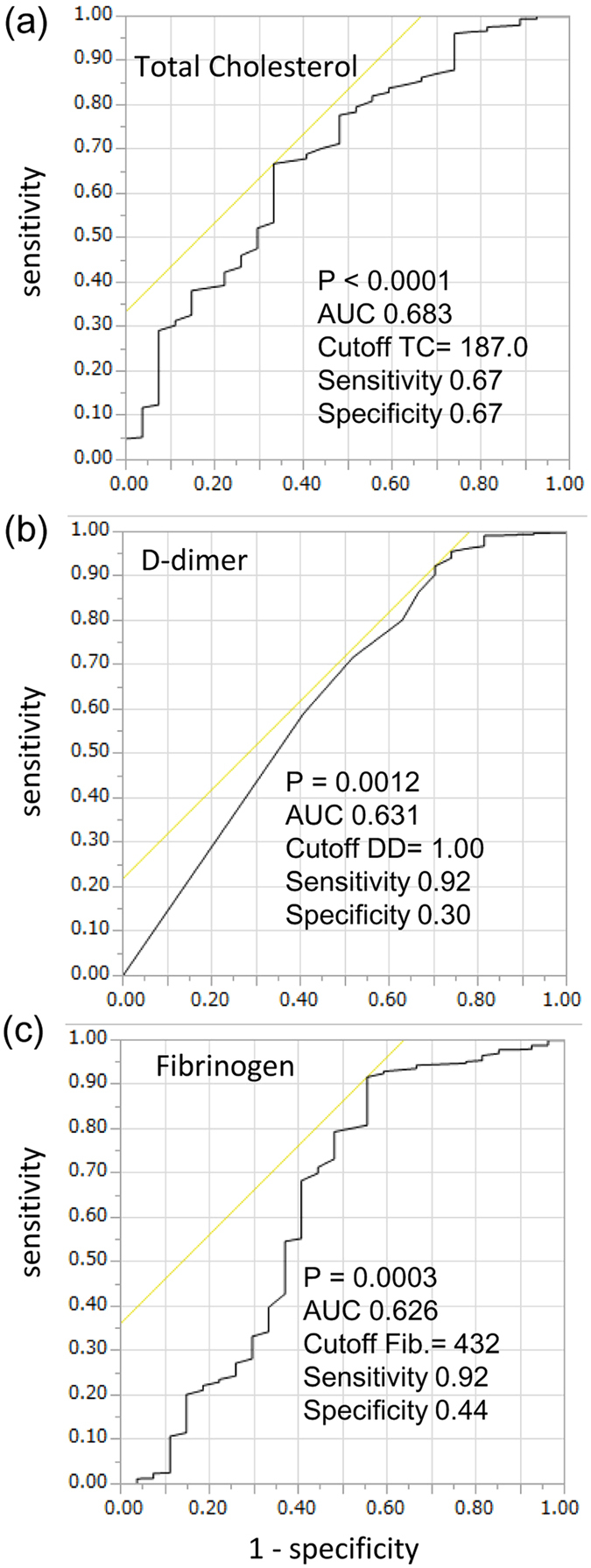
Determination of cut-off values for circulating cholesterol, D-dimer, and fibrinogen concentrations for discriminating subjects who died of respiratory failure by the end of the follow-up period. Receiver operating characteristic (ROC) curve analysis was performed to determine the cut-off values of cholesterol (**a**), D-dimer (**b**), and fibrinogen concentrations (**c**) for discriminating subjects who had died of respiratory failure. The area under the curve (AUC) was 0.683, 0.631, and 0.626, and the cut-off value was 187 mg/dL, 1.0 μg/mL, and 432 mg/dL, with a sensitivity of 0.67, 0.92, and 0.92, and a specificity of 0.67, 0.30, and 0.44 for cholesterol, D-dimer, and fibrinogen, respectively.

The cut-off values for TC, D-D, and fibrinogen for predicting death by respiratory failure was determined using ROC analysis (Fig. 1). The cut-off values for TC, D-D, and fibrinogen were 187 mg/dL (AUC: 0.683), 1.00 μg/mL (AUC: 0.631), and 432 mg/dL (AUC: 0.626), respectively.

**Figure 2:**
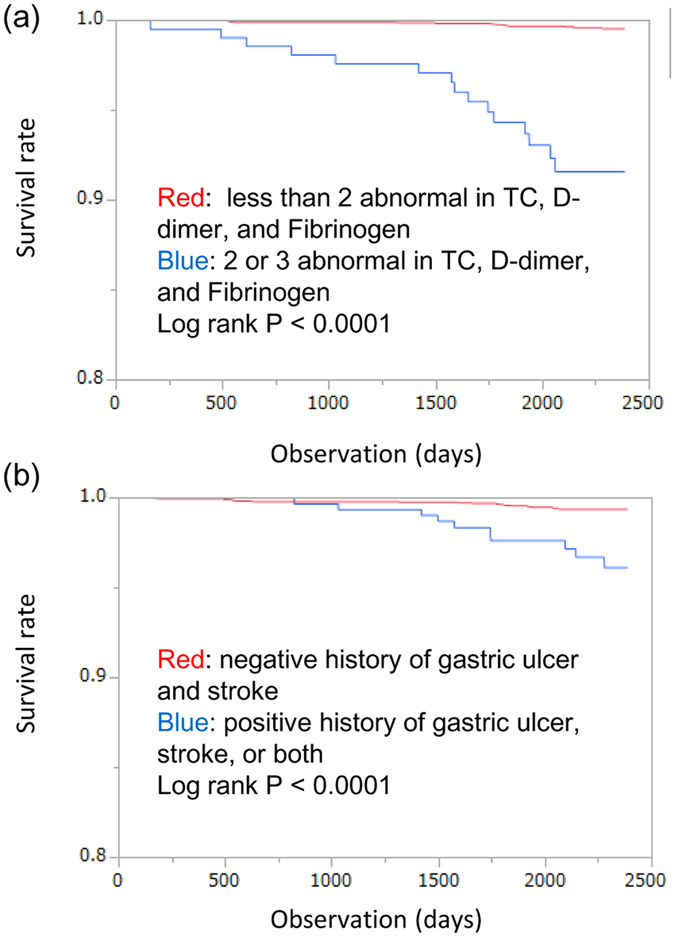
Kaplan-Meier survival curve for mortality from respiratory failure according to grade of risk. Survival curves for mortality due to respiratory failure relative to the stratified risk are shown for cholesterol (TC), D-dimer (D-D), and fibrinogen concentrations (**a**), and disease history (**b**). (**a**) Subjects with two or more abnormal values in the TC, D-D, and fibrinogen were placed in the high-risk group and remainder in the non-high risk group. (**b**) Subjects with a history of stroke or gastric ulcer were placed in the high-risk group and remainder in the non-high risk group. The survival rates of the high-risk groups were significantly lower than those of the non-high risk group (**a**,**b**). Blue lines, high-risk groups; red lines, non-high risk groups.

The usefulness of the laboratory data, including TC, D-D, and fibrinogen and the history of diseases such as stroke and gastric ulcer, was determined using Kaplan-Meier analysis, because these were independent risk factors for predicting respiratory mortality. Subjects who had two or more abnormalities in the TC, D-D, and fibrinogen were placed in the high-risk group, and the remaining subjects were placed in the non-high-risk group (Fig. 2a). Subjects who had a history of stroke and gastric ulcer were placed in the high-risk group, and remaining subjects were placed in the non-high risk group (Fig. 2b). As shown in Fig. 2, subjects in the high-risk groups had significantly lower survival rates than those in the non-high risk groups.

### Addition of disease history and laboratory data to the basic parameters in predicting respiratory death

**Figure 3:**
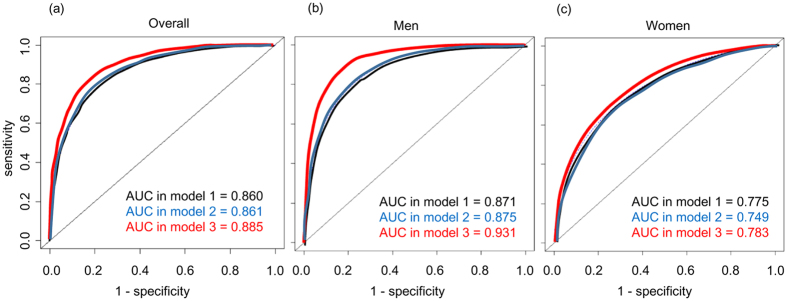
Receiver operating characteristic (ROC) curve analyses for predicting respiratory death by Cox proportional hazard analysis. ROC curves for predicting respiratory death by Cox proportional hazard analyses were plotted in all (**a**), male (**b**), and female (**c**) subjects. Variables in each model were as follows: model 1 (black curve), age, sex and BMI; model 2 (blue curve), age, sex, BMI, history of stroke and gastric ulcer; and model 3 (red curve), age, sex, BMI, history of stroke and gastric ulcer, and total cholesterol, D-dimer, and fibrinogen concentrations. Values of area under the curve (AUC) are indicated in the figure. Values of 95% confidence interval of AUC are shown in Table 3 of this manuscript.

Because advanced age, male sex, and low BMI were strong risk factors for death by respiratory failure, we evaluated whether including the disease history (stroke and gastric ulcer) and laboratory data (TC, D-D, and fibrinogen) with the basic parameters (age, sex, and BMI) improved the prediction of respiratory death in the Cox proportional hazard analysis by using C-statistics. In overall subjects, area under curves (AUCs) of receiver operator characteristics (ROC) curves were not significantly different among model 1, model 2, and model 3 (Fig. 3a and Table 3). However, since respiratory death occurred more frequently in men than women, the differences of AUCs were separately analyzed in men and women. The addition of laboratory data (TC, D-D, and fibrinogen) significantly improved the AUC in model 3 compared to that in models 1 and 2 in men (Fig. 3b and Table 3), but not in women (Fig. 3c and Table 3).

Although C statistics has been the common method for quantifying the improvement in prediction, some studies reported the limitations of this method including difficulty in interpretation of small magnitude changes and lack of clinical relevance^3^,^4^. Therefore, we used integrated discrimination improvement (IDI) and net reclassification improvement (NRI) to assess if the addition of information about disease history and laboratory data improved the prediction of respiratory death^4^. The addition of the disease history significantly improved the IDI and NRI in model 2 compared to model 1 (Table 3). Furthermore, the addition of laboratory data (TC, D-D, and fibrinogen) significantly improved the NRI, and IDI (Table 3) in model 3 compared to models 1 and 2.

### Association between serum pepsinogen concentration and death by respiratory failure

We also measured the *Helicobacter pylori* antibody, pepsinogen I (PEP1), and pepsinogen II (PEP2) concentrations in the study population. The PEP1, PEP2, and PEP1/PEP2 were not normally distributed; therefore, the data were log-transformed for analysis. Many subjects were positive for *H. pylori* antibody (Table 4A). The log PEP1 and log PEP1/PEP2 in the group DR and group DoR were significantly lower than those in group A. The log PEP2 did not differ significantly between the groups (Table 4A). The PEP1 and PEP1/PEP2 were inversely associated with age [PEP1 (ng/mL) = 72.33 − 0.36 × age, *P* < 0.0001; PEP1 (ng/mL)/PEP2 (ng/mL) = 5.95 − 0.045 × age, *P* < 0.0001], and subjects in groups DR and DoR were significantly older than subjects in group A (Table 1). The PEP1 and PEP1/PEP2 were log-transformed into the percent predicted values according to subject age, and the differences were reanalyzed between the groups (Table 4B). The log PEP1 % predicted and log PEP1/PEP2 % predicted in group DR and DoR was significantly lower than that in group A (Table 4B).

## Discussion

This study demonstrated that a history of stroke or gastric ulcer, lower BMI and TC concentration, and higher D-D and fibrinogen concentrations were associated with death by respiratory failure. Decreased BMI was previously reported to be a risk for respiratory mortality in a Japanese elderly population^5^. However, the present study is the first to demonstrate the usefulness of information concerning the nutritional condition, basal disease, and laboratory data for predicting mortality by respiratory death in a general population.

The BMI is a simple index representing the nutritional condition^6^. In patients with respiratory disease, a lower BMI is associated with a poorer prognosis^7^,^8^,^9^,^10^. Reportedly, the relationship between mortality due to all causes and the BMI exhibits a U-shape in the general population^11^. This means that individuals with a very low or very high BMI have a poorer prognosis, and individuals with an optimal BMI tend to live longer. In Japanese elderly individuals, those with a low BMI had the highest rate of mortality due to respiratory disease^5^. However, another study in a Japanese general population demonstrated that the relationship between mortality due to pneumonia and BMI was U-shaped^12^. In the present study, 3253 subjects from a healthy general population were followed for approximately 7 years, and 27 subjects died due to respiratory disease. We suspect the subpopulation of respiratory deaths was too small to show the expected U-shaped relationship between respiratory mortality and BMI in this study population. However, low BMI, which represents poor nutritional condition, was associated with a poor outcome in the form of respiratory failure.

In the present study, we found that the presence of other diseases historically predicted mortality by respiratory failure. Stroke is a known risk for pneumonia because it impairs function in the central nerve system, leading to swallowing disturbances, and induces poor immune status in the elderly^13^. In a 5-year follow-up of stroke patients, the most common cause of hospital re-admission was respiratory disease, including pneumonia^14^. However, in the cohort for the BODE study, having stroke as a comorbidity was not a significant risk for death in COPD patients^15^. To date, there are no reports showing a relationship between stroke and respiratory death in a general population. Thus, the significance of this study is that we successfully demonstrate this relationship in a Japanese general population.

It is unknown whether gastric ulceration itself induces respiratory diseases such as pneumonia. There are no known reports showing a relationship between gastric ulcers and respiratory disease in the general population. In the BODE study, having gastric ulcer as a comorbidity was demonstrated as a risk for death in COPD patients^15^. In patients with COPD comorbid with gastroesophageal reflux disease (GERD), a lower prescription rate of antacids was associated with a higher frequency of COPD exacerbation^16^,^17^. Thus, excess gastric acid and reflux may exacerbate COPD. One possible explanation for the association between respiratory death and a history of gastric ulceration in this population is that excess secretion of gastric acid may cause reflux of gastric acid, which may cause chronic lower respiratory damage.

An alternative explanation is also plausible. Many patients with gastric ulceration are prescribed antacids such as proton pump inhibitors (PPIs). Elevation of the gastric pH by PPIs promotes bacterial growth in the stomach, and PPI use is thought to increase the risk of community-acquired and hospital-acquired pneumonia^18^. In the present study, it is unclear how many subjects were taking antacids including PPIs. As shown in Table 4, the serum PEP1 and PEP1/PEP2 levels were significantly lower in subjects who died of respiratory failure than the levels in subjects who remained alive. Lower PEP1 and PEP1/PEP2 levels are markers of atrophic gastritis^19^. This suggests that the gastric mucosa in the subjects who died of respiratory failure tended to be more atrophied, in part because antacids were not prescribed sufficiently for the subjects. In contrast to COPD patients with GERD, lower serum PEP1 and PEP1/PEP2 levels suggest that gastric pH in the elderly who died of respiratory failure may be elevated owing to mucosal atrophy. This may result in abnormal bacterial growth in the stomach, and may further increase the incidence of respiratory failure caused by lower respiratory infection due to reflux of gastric contents. Regardless of the mechanism, subjects with a history of gastric ulcers should be carefully and continuously monitored in order to determine the cause of the relationship between respiratory death and gastric ulceration.

Circulating cholesterol accumulates into atherosclerotic lesions, and these cholesterol plaques subsequently cause cardiovascular disease^20^. Smith and colleagues reported that the association between the serum cholesterol concentration and all causes of mortality exhibited a U-shape in the general population^21^. In addition, they demonstrated that high and low cholesterol concentrations were associated with higher cardiovascular mortality and higher mortality caused by neoplastic and respiratory diseases, respectively^21^. By contrast, although Iribarren and colleagues reported that a low cholesterol level was associated with hospitalization for pneumonia, the only pulmonary disease that showed a significant association between low cholesterol and mortality was COPD^22^. In this study, low TC was associated with mortality caused by respiratory failure. Low TC concentration may indicate a poor nutritional condition or immune capacity in subjects from a general population.

Fibrinogen and D-D are markers for coagulation and fibrinolysis, respectively^23^. A poor survival rate has been reported in pneumonia patients and COPD patients with a high D-D concentration^24^,^25^. In addition, the association between elevated D-D and the risk of all-cause mortality was demonstrated in a general population^26^,^27^. Recently, fibrinogen has been described as an inflammatory biomarker^28^, and a higher fibrinogen concentration was associated with a higher all-cause mortality rate and higher mortality due to cardiovascular disease in a general population, but not due to respiratory disease^27^. However, we demonstrated a significant inverse relationship between the plasma fibrinogen concentration and the FEV1 in this population^29^. In the ECLIPSE Study, fibrinogen was demonstrated to be a biomarker for death and exacerbation in COPD patients^30^. Notably, the present study is the first known report showing a significant relationship between coagulo-fibrinolytic markers and the risk of mortality due to respiratory failure in a general population.

The present study has several other limitations. First, because the blood was sampled one time, we could not determine if the measured values represent the steady state condition. Second, it is unclear whether the subjects who died of respiratory failure had a similar socioeconomic background compared with the remaining subjects. Third, the number of subjects who died of respiratory failure by the end of the follow-up period was too few to allow analysis according to the specific respiratory disease.

In conclusion, we showed that subjects who were older, male, underweight, hypocholesterolemic, had a hypercoagulo-fibrinolytic status and a history of stroke or gastric ulcer had a higher risk of mortality due to respiratory failure in a prospective cohort of the Japanese general population. Future studies are needed to investigate whether interventions such as vaccination, prevention of aspiration such as oral care, and use of antacids or prokinetic agents can reduce the mortality due to respiratory failure in the high-risk population identified in the present study.

## Methods

### Study population

This study was part of the Molecular Epidemiological Study, which was supported by the Regional Characteristics of 21^st^ Century Centers of Excellence (COE) Program and the Global COE Program in Japan. All procedures were approved by the ethics committee of the Yamagata University School of Medicine, and was conducted according to the recommendations of the Declaration of Helsinki^2^,^31^. Written informed consent was provided by all participants. Subject enrollment (1500 men; 1753 women) has been described previously^2^,^31^. Subjects’ deaths were confirmed by their doctors, and the death certificates written by the doctors were reported to the local government, Takahata town, by their families. The information about their primary cause of death was summarized according death certificates in the database. We obtained the database of death certificates from Takahata government, and subjects’ deaths through the end of 2010 were reviewed by examining the database^2^. In the database, the information on comorbidities related to the primary cause of death was not available.

### Measurements

Blood pressure, spirometry, and blood sampling were performed as previously described in the Molecular Epidemiological Study^2^,^31^. Serum levels of antibody against *H. pylori*, PEP1 and PEP2 were measured by another laboratory (BML, Inc., Tokyo, Japan). Since PEP1 and PEP1/PEP2 levels were inversely associated with age, we obtained the liner regression formula predicting the PEP1 and PEP1/PEP2 values according to age. Percent predicted of PEP1 and PEP1/PEP2 levels were obtained by dividing the actual values by predicted values, and these percent predicted (age-adjusted) values were used for the analyses in order to compare the levels of PEP1 and PEP1/PEP2 among the population whose mean ages were significantly different. In addition, because PEP1, PEP2, and PEP1/PEP2 were not normally distributed, these were log-transformed.

### Statistical analysis

For continuous variables, data are presented as the mean (standard deviation [SD]). The χ^2^ test was performed to evaluate differences in proportion. One-way analysis of variance was used for multiple comparisons, followed by the Tukey test. Cox proportional hazard analyses were performed to assess potential independent associations between the variables and mortality caused by respiratory failure. The results of the Cox proportional hazard analyses are presented as hazard ratios (HR) with 95% confidence intervals (CI) according to a 1-SD increase of each variable or difference in positivity in the disease history.

ROC curve analysis was performed to determine the cut-offs for factors discriminating death by respiratory failure. The values that maximized the sum of sensitivity and specificity were determined as the cut-offs. The Kaplan-Meier method with log-rank test was used to examine differences in the rate of mortality due to respiratory failure according to the stratified risk. The AUC was used as a measure of the predictive accuracy of disease history and laboratory data for death by respiratory failure. The NRI and the IDI were calculated to measure the magnitude of improvement upon correct reclassification and the sensitivity after including information on disease history and laboratory data in the model^2^. Statistical analyses were performed using JMP version 11 software (SAS Institute Inc., Cary, NC, USA) or R 3.0.2 with additional packages (survC1, risksetROC and survIDINRI).

## Additional Information

**How to cite this article**: Kobayashi, M. *et al*. Predictors for mortality from respiratory failure in a general population. *Sci. Rep.* **6**, 26053; doi: 10.1038/srep26053 (2016).

## Figures and Tables

**Table 1:** Subject characteristics in each group.

	Group A	Group DR	Group DoR	P
Age, years	61.9 (10.3)	72.9 (7.0)***	71.3 (8.5)***	<0.0001
Female, %	54.9	25.9**	29***	<0.0001
Brinkman Index	190.6 (383.4)	367.7 (478.0)	320.7 (524.0)**	0.0008
BMI, kg/m^2^	23.5 (3.2)	20.9 (2.7)***	23.1 (3.5)^##^	<0.0001
Systolic BP, mm Hg	134.2 (15.9)	135.8 (14.6)	137.3 (18.9)	0.1398
%FVC	98.8 (14.4)	95.2 (13.1)	95.1 (20.7)*	0.0199
%FEV1	98.0 (16.2)	88.3 (21.7)**	92.9 (23.6)**	0.0001
FEV1/FVC < 0·7, %	9.8	37.4***	21.0**	<0.0001
RBC, ×10^4^/mm^3^	441.4 (43.7)	425.7 (42.9)	429.3 (53.2)*	0.005
Hb, g/dL	13.7 (1.5)	13.6 (1.3)	13.6 (1.8)	0.7152
Ht, %	41.1 (4·2)	40.6 (3.9)	40.8 (5.3)	0.7041
Albumin, g/dL	4.5 (0.2)	4.4 (0.6)	4.5 (0.3)	0.0538
AST, U/L	24.5 (12.2)	30.7 (11.9)*	27.3 (11.6)	0.008
ALT,U/L	23.4 (14.0)	25.7 (17.2)	21.9 (10.6)	0.3763
BUN, mg/dL	16.2 (4.6)	16.5 (5.5)	17.0 (4.1)	0.1916
sCr, mg/dL	0.67 (0.22)	0.76 (0.21)	0.75 (0.19)*	0.0003
UA, mg/dL	5.1 (1.3)	5.5 (1.0)	5.5 (1.3)**	0.0031
HbA1c, %	5.3 (0.7)	5.5 (1.2)	5.3 (0.7)	0.1095
TC, mg/dL	201.1 (31.7)	177.6 (35.9)***	194.5 (40.2)^#^	0.0001
TG, mg/dL	107.7 (64.9)	91.0 (39.5)	105.9 (72.0)**	0.4007
sFe, μg/dL	105.3 (36.4)	103.2 (37.3)	111.5 (40.9)	0.2365
log Hcy	1.02 (0.13)	1.12 (0.19)***	1.11 (0.15)***	<0.0001
Adiponectin, μg/mL	10.0 (5.5)	11.3 (7.0)	12.0 (7.5)**	0.0009
Renin, ng/mL/hour	1.53 (2.18)	2.68 (3.49)*	2.30 (2.96)**	<0.0001
D-D, ng/mL	0.66 (0.43)	1.12 (1.05)***	0.77 (0.43)^###,^*	<0.0001
Fibrinogen, mg/dL	332.5 (69.3)	381.7 (132.2)***	358.5 (83.8)***	<0.0001
P.H. heart dis., %	4.06	3.7	3	0.8648
P.H. stroke, %	0.93	7.41	1	0.0031
P.H. gastric ulcer, %	8	33.33	7	<0.0001
P.H. duodenal ulcer, %	5.02	3.7	8	0.3898
P.H. Respir. Dis., %	2.34	7.41	6	0.0177
P.H. cancers, %	2.59	14.81	2.0	0.0004

Subjects were divided into three groups according to the outcome at the end of follow-up as follow: Group A included subjects who were alive at the end of the follow-up period; Group DR included subjects who died of respiratory failure; and Group DoR included subjects who died of diseases other than respiratory failure. The Brinkman Index was unavailable in 267 male and 63 female subjects. The Hcy concentration was unavailable in 29 male and 94 female subjects.

Values are the mean (standard deviation [SD]) or percentage. Differences were evaluated by the Chi-square test or analysis of variance, followed by the Tukey test.

*P < 0.05, **P < 0.01, and ***P < 0.0001 compared with group A. ^#^*P* < 0.05, ^##^*P* < 0.01, and ^###^*P* < 0.0001 compared with group DR.

BMI: body mass index; BP: blood pressure; FVC: forced vital capacity; FEV1: forced expiratory volume in 1 s; RBC: red blood cell count; Hb: hemoglobin; Ht: hematocrit; AST: aspartate aminotransferase; ALT: alanine aminotransferase; BUN: blood urea nitrogen; sCr: serum creatinine; UA: uric acid; HbA1c: hemoglobin A1c; TG: triglyceride; TC: total cholesterol; sFe: serum iron; Hcy: homocysteine; D-D: D-dimer; P.H.: past disease history of; Dis.: disease; Respir.: respiratory.

**Table 2:** Cox proportional hazard analyses of factors predictive for death by respiratory failure.

	HR	95% CI	P
Age*	3.58	2.00–6.84	<0.0001
Male gender	8.16	2.81–26.31	<0.0001
BMI	0.44	0.27–0.69	0.0003
TC	0.57	0.37–0.87	0.0102
Hcy	1.12	0.91–1.23	0.2077
D-D	1.27	1.08–1.38	0.0074
Fibrinogen	1.71	1.23–2.25	0.0018
P.H. stroke**	7.74	1.19–28.78	0.0352
P.H. gastric ulcer	3.84	1.51–9.07	0.0058
P.H. cancer	2.98	0.83–8.24	0.0873
P.H. Respir. Dis.	1.01	0.16–3.56	0.9902

HR: hazard ratio; CI: confidence interval; BMI: body mass index; TC: total cholesterol; Hcy: homocysteine; D-D: D-dimer; P.H.: past disease history of; Respir.: respiratory; Dis.: disease; SD: standard deviation.

^*^Hazard ratios for age, BMI, TC, Hcy, and D-D were analyzed per 1-SD increase in the respective parameters.

^**^Hazard ratios for a positive history of each comorbidity was determined compared with a negative history of the respective comorbidities.

**Table 3:** Statistical model improvement following the addition of disease history (stroke and gastric ulcer) and laboratory data (cholesterol, D-dimer, and fibrinogen concentration) in predicting mortality due to respiratory failure.

	Model 1	Model 2	Model 3
AUC overall	0.860 (0.794–0.927)	0.861 (0.790–0.931)	0.885 (0.814–0.955)
AUC men	0.871 (0.806–0.935)	0.875 (0.801–0.948)	0.931 (0.877–0.985)^*,#^
AUC women	0.755 (0.553–0.957)	0.749 (0.557–0.943)	0.783 (0.596–0.971)
NRI^a^	–	0.277 (0.029–0.468)*	0.415 (0.181–0.624)**
NRI^b^	–	–	0.328 (0.018–0.541)^#^
IDI^a^	–	0.058 (0.014–0.211)**	0.093 (0.052–0.311)**
IDI^b^	–	–	0.035 (0.004–0.204)^#^

Letters indicate the hazard ratio compared with ^a^ model 1 and ^b^ model 2. Variables in each model were as follows: model 1, age, sex and BMI; model 2, age, sex, BMI, history of stroke and gastric ulcer; and model 3, age, sex, BMI, history of stroke and gastric ulcer, and total cholesterol, D-dimer, and fibrinogen concentrations.

The improvement in the hazard ratio in the Cox proportional hazard test was evaluated. Data are presented as the hazard ratio (95% confidence interval). *P < 0.05 and **P < 0.01 vs. model 1; ^#^*P* < 0.05 vs. model 2.

AUC, area under the ROC curve; ROC, receiver operator characteristics; NRI, net reclassification improvement; IDI, integrated discrimination improvement.

**Table 4:** Incidence of *Helicobacter pylori* antibodies, log-transformed serum concentrations of pepsinogen I and II, and ratio of pepsinogen I/II in the all patients.

	Group A (n = 3117)	Group DR (n = 27)	Group DoR (n = 100)	P
**A**				
Positive for *H. pylori* antibody	2152 (69.0%)	21 (77.8%)	74 (74.0%)	0.715
Log_10_ PEP 1	1.61 (0.31)	1.45 (0.40)*	1.46 (0.42)*	<0.00001
Log_10_ PEP 2	2.74 (0.64)	2.79 (0.62)	2.73 (0.59)	0.8827
Log_10_ PEP 1/PEP 2	0.42 (0.28)	0.24 (0.32)*	0.28 (0.33)*	<0.00001
**B**				
Log_10_%PEP 1	1.91 (0.31)	1.79 (0.39)*	1.79 (0.42)*	0.0002
Log_10_%PEP 1/PEP 2	1.92 (0.27)	1.82 (0.32)*	1.84 (0.33)*	0.0018

Subjects were divided into three groups according to the outcome at the end of follow-up as follow: Group A included subjects who were alive at the end of the follow-up period; Group DR included subjects who died of respiratory failure; and Group DoR included subjects who died of diseases other than respiratory failure. Because PEP1, PEP2, and PEP1/PEP2 were not normally distributed, the data were log-transformed.

(**A**). The log PEP1 and PEP1/PEP2 in groups DR and DoR were significantly lower than in group A.

(**B**) PEP1 and PEP1/PEP2 were inversely associated with the age [PEP1 (ng/mL) = 72.33 − 0.36 × age, *P* < 0.0001; PEP1 (ng/mL)/PEP2 (ng/mL) = 5.95 − 0.045 × age, *P*  < 0.0001], and subjects in groups DR and DoR were significantly older than those in group A. The PEP1 and PEP1/PEP2 were log-transformed into the percentage predicted value according to age.

Values are presented as the number (%) or mean (standard deviation [SD]). Differences were evaluated by analysis of variance, followed by the Tukey test. *P < 0.05 vs. group A in the Tukey test

H., Helicobacter; PEP, pepsinogen.
